# Water Infusions of Motherwort (*Leonurus cardiaca*) as a Source of Chlorogenic Acid and Antioxidant Properties

**DOI:** 10.3390/foods15111846

**Published:** 2026-05-23

**Authors:** Anna Przybylska, Anna Maria Proszowska, Marcin Koba, Magdalena Woźniak, Łukasz Rzepiński, Włodzimierz Gniłka

**Affiliations:** 1 Department of Toxicology and Bromatology, Faculty of Pharmacy, Ludwik Rydygier Collegium Medicum in Bydgoszcz, Nicolaus Copernicus University in Torun, 85-094 Bydgoszcz, Polandannaproszowska@cm.umk.pl (A.M.P.); 2 Department of Medical Cosmetology, Faculty of Pharmacy, Ludwik Rydygier Collegium Medicum in Bydgoszcz Nicolaus Copernicus University in Torun, 85-094 Bydgoszcz, Poland; 3 Department of Neurology, 10th Military Research Hospital and Polyclinic, 85-681 Bydgoszcz, Poland; 4 Department of Clinical Medicine, Faculty of Medicine, University of Science and Technology, 85-796 Bydgoszcz, Poland; 5 Department of General and Minimally Invasive Surgery, Dr. J. Biziel University Hospital No. 2, Ludwik Rydygier Collegium Medicum in Bydgoszcz, Nicolaus Copernicus University in Toruń, 85-168 Bydgoszcz, Poland; 6 Sanitas—Neurology Outpatient Clinic, 85-010 Bydgoszcz, Poland

**Keywords:** chlorogenic acid, *Leonurus cardiaca*, antioxidant properties, capillary electrophoresis, phenols, water infusions

## Abstract

Background: It is important to note that motherwort (*Leonurus cardiaca*) has especially calming properties, partly due to its chlorogenic acid (CLA) content. While the concentration of this acid in alcoholic or water–alcoholic extracts has been determined in the literature, there is no knowledge about the content of CLA in water extracts. Therefore, the aim of this study was primarily to assess the CLA content in infusions prepared in four different ways and to estimate the actual CLA intake with these infusions. An additionally aim was to assess the content of phenols and their antioxidant properties. Methods: A capillary electrophoresis device equipped with a DAD detector was used to determine CLA in dried motherwort herb, and the DPPH test and the Folin–Ciocâlteu method were used to determine antioxidant properties. Results: The median CLA content in the infusions was 527.26 µg/100 mL. In turn, the total phenol content per glass was 32.88 mg. The mean median DPPH test expressed as Trolox equivalent was 33.65 mg/L TE. Conclusions: The method of preparing infusions affects the content of CLA and phenols in water infusions. However, these changes did not significantly affect the antioxidant properties of the prepared extracts.

## 1. Introduction

In recent years, the “eco” trend has been noticeable, which has translated into an increase in interest in natural food, including herbal products. It is estimated that global sales of herbal products amounted to USD 220 billion and this trend is growing [1]. The consumption of herbs and dietary supplements is observed in people suffering from depression or cardiovascular diseases such as hyperlipidemia, atrial fibrillation and heart failure [1,2]. According to the World Health Organization (WHO), approximately 300 million people suffer from depression. As many as 60–90% of medical interventions are closely related to stressful environmental conditions, and as many as 80% of the population prefer the use of sedatives and anti-anxiety agents in the form of herbal products [2].

**Figure 1:**
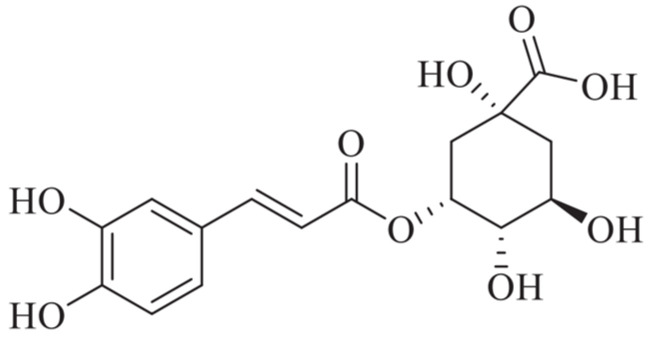
Chemical structure of chlorogenic acid.

One of the biologically active compounds with antidepressant properties is chlorogenic acid (CLA) [3]. Studies show that CLA increases the levels of serotonin and dopamine in the serum of depressed rats, thus demonstrating antidepressant effects, but it also has anxiolytic effects [3,4]. CLA (3-*O*-Caffeoylquinic acid) is a secondary metabolite widely distributed in the plant world. The structure is shown in Figure 1. In vitro and in vivo studies have shown that the main pharmacological effects of CLA are antioxidant, anti-inflammatory, antibacterial, antiviral, hypoglycemic, lipid-lowering, anticardiovascular, antimutagenic, anticancer and immunomodulatory [5,6]. Some of the CLA that reaches the stomach and/or small intestine is absorbed intact. The current state of knowledge indicates that approximately one third of ingested CLA is absorbed in the digestive tract and enters the bloodstream [7]. Consumption of CLA is associated with reduced risk cardiovascular diseases but also diabetes [5]. This effect is due to its antioxidant and anti-inflammatory properties [6]. Moreover, it also affects glucose and lipid metabolism by increasing mRNA expression of PPAR-α and hepatic X receptor α as key mediators of lipid metabolism and inhibition of glucose-6-phosphatase activity that reduces glucose production in the liver [5]. Studies also indicate that the consumption of products rich in CLA by overweight consumers causes increase in adiponectin and high-density lipoprotein cholesterol concentration [6].

Motherwort (*Leonurus cardiaca* L.) is an herb in the *Lamiaceae* family. This plant comes from Asia, but due to its medicinal use, the plant is now spread all over the world [8]. The European Pharmacopoeia recommends the use of whole or crushed, dried and flowering aerial parts of the plant containing not less than 0.2% of flavonoids expressed as hyperoside. Initially, motherwort preparations were used as cardiotonic agents regulating the heart rhythm, in angina pectoris, circulatory system neuroses and initial stages of hypertension. Currently, it is used in cardiosclerosis, increased blood pressure, angina pectoris, myocarditis and excessive nervous excitability, especially when it is associated with menopause in women and prostate enlargement in men. Up to now, about 270 substances have been isolated from motherwort herb [2]. The pharmacological activity of *L. cardiaca* results mainly from the presence of furan diterpenes (labdane), alkaloids (stahydrin), sterols, iridoids, flavonoids and ursolic acid, but also CLA [9]. Clinical studies indicate that motherwort herbs have hypotensive, cardiotonic and sedative effects as well as cause antibacterial and anti-inflammatory effects [8,9].

The aim of this study was primarily to assess the CLA content in infusions using capillary electrophoresis and then to estimate the actual CLA intake by consumers consuming these infusions. Additionally, the aim was to assess the content of phenols in water infusions of *Leonurus cardiaca* and their antioxidant properties.

## 2. Materials and Methods

### 2.1. Samples

*L. cardiaca* was purchased in herbal stores in Poland. The samples came three leading Polish producers of herbal products (Table 1 and Table 2). Each manufacturer recommends a different way of preparing water infusions. Four water infusions were prepared from each of them, differing in the preparation method (see Table 1).

### 2.2. Preparation of Water Infusions

Samples of water infusions of plant raw material were prepared as follows: 1.0 g of raw material with 50 mL of water (100 °C), brewed for 10 min (extraction method A); 1.0 g of raw material with 50 mL of water (100 °C), brewed for 20 min (extraction method B); 0.6 g of raw material with 50 mL of water (100 °C), brewed for 10 min (extraction method C); 0.6 g of raw material with 50 mL of water (100 °C), brewed for 20 min (extraction method D). The infusions prepared in this way were filtered through paper and then through a Chromafil Xtra PES-45/25 syringe filter (Mecherey-Nagel, Düren, Germany).

### 2.3. Determination of the Total Phenolic Content

The total content of polyphenolic compounds in the aqueous extract of the samples was tested according to previous methods with minor modifications [10]. A volume of 0.5 mL of aqueous infusion was taken and added to a 5 mL test tube. Volumes of 0.5 mL of Folin–Ciocâlteu’s (FC, FC:H_2_O, 1:2) solution, 1.0 mL of 20% Na_2_CO_3_ and 1.0 mL of water were added. Then, it was incubated in the dark for 30 min and the absorbance was measured at a wavelength of 700 nm against the reference solution. The reference solution was prepared in a similar way, replacing 0.5 mL of the aqueous infusion with water. Six solutions of gallic acid (GAL, Sigma-Aldrich, St. Louis, MO, USA) with concentrations of 6.25–50.00 mg/L were prepared. The standard curve shows the dependence of the absorbance value of GAL on its concentration. The results obtained were expressed in terms of GAL equivalent (mg GAL/100mL). The prepared standard curve was characterized by high linearity, which was confirmed by the high value of the correlation coefficient R2 = 0.995 (y = 0.0176x + 0.0018).

### 2.4. Determination of the Chlorogenic Acid Content

The capillary electrophoresis instrument equipped with diode array detector was used for analysis. The chlorogenic acid was separated using a fused silica capillary column of 60 cm total length and 50 cm effective length (75 I.D., 375 O.D.). The background electrolyte (BGE) consisting of 20 mM tetraborate buffer was used. The pH of the buffer was 9.40. Between each injection, the capillary was rinsed with 0.1 M NaOH for 1 min, then rinsed with the solution with deionized water for 1 min, and finally, the capillary was rinsed with borate buffer for 1 min. The samples were hydrodynamically injected for 5 s, the system was operated under positive voltage (30 kV) and the cassette temperature was 25 °C. Electropherograms were recorded at 350 nm. Six solutions of chlorogenic acid (CLA, Sigma Aldrich, USA) with concentrations of 5.0–25.0 mg/L were prepared. The standard curve shows the dependence of the peak area value of CLA on its concentration. The prepared standard curve was characterized by high linearity, which was confirmed by the high value of the correlation coefficient R2 = 0.997 (y = 902.09x + 1976.4), while the CE analytical method was used for the chlorogenic acid assay characterized by LOD and LOQ on the levels of 2.34 and 10.50 µg/mL, respectively, with precision defined as RSD% amounting to 2.71 and accuracy on the level 0.25 characterized by the STD% value.

### 2.5. Determination of the Antioxidant Activity of Aqueous Extracts Using the DPPH Radical

In order to determine the antioxidant activity of aqueous extracts of motherwort herb, previous methods were used with slight modifications [11,12]. The DPPH (2,2-difenylo-1-pikrylohydrazyl) solution (100 µM) was prepared by weighing 4 mg of DPPH (Sigma Aldrich, USA) and dissolving it in 100 mL of methanol (POCH, Gliwice, Poland). A volume of 0.5 mL of aqueous extract/standard solution (TE; Trolox) was taken and added to 2 mL of DPPH solution. Absorbance was measured at 517 nm against a methanol reference after 30 min of incubation in the dark. Six solutions of Trolox (TE, (+-)-hydroxy-2,5,7,8-tetramethyl-chroman-2-carboxylic acid, Sigma Aldrich, USA) with concentrations of 1.25–8.00 mg/L were prepared. The standard curve shows the dependence of the absorbance value of Trolox on its concentration. The results obtained were expressed in terms of Trolox equivalent (mg TE/100 mL). The prepared standard curve was characterized by high linearity, which was confirmed by the high value of the correlation coefficient R2 = 0.939 (y = −0.0583x + 0.4491). Additionally, the percentage ability of the aqueous extract to reduce the DPPH radical was calculated:

% inhibition = [(A − Ab)/A] × 100

A—control sample absorbance (100 µM methanol solution of DPPH• whose absorbance was measured at the beginning and at the end of the experiment).

Ab—mean value of absorbance of the tested aqueous extract.

### 2.6. Statistical Analysis

The results obtained during this research were subjected to statistical analysis using the Statistica ver 13 program (StatSoft, Tulsa, OK, USA). The normality of the distribution of variables was checked using the Shapiro–Wilk test (*p* < 0.05). Differences between parameters values were calculated using a non-parametric test Kruskal–Wallis. Spearman’s correlation was also used to determine the relationship between significant variables.

## 3. Results

This study assessed the influence of the method of preparing water infusions from dried motherwort herb on the content of chlorogenic acid and total phenols content. The antioxidant properties of the prepared infusions were also evaluated using the DPPH test. The results of the chlorogenic acid determinations are summarized in the Table 3. Please note that each manufacturer recommends a different method of preparing infusions. For uniformity, the research was conducted using four different methods of preparing infusions.

The research shows that the method of preparing water infusion of motherwort herb affects the content of chlorogenic acid in it. In the 128 infusions we analyzed, the average (median) content of chlorogenic acid was 527.26 µg/100mL and the range was between 197.94–899.01 μg/100 mL. Due to the fact that consumers take into account the volume of the glass when preparing infusions, the results are presented per glass (250 mL). Therefore, the results showed that when weighing 1.0 g of raw material and brewing for 10 or 20 min, the CLA content was 1.60 and 1.56 mg/glass. In turn, the median CLA content per glass, regardless of the method of preparing the infusions, was 1.32 mg/glass. The highest CLA content was found for the infusion prepared by weighing 1 g of the raw material and pouring 50 mL and infusing it for 10 min. Increasing the brewing time to 20 min did not increase the degree of leaching of CLA into the aqueous solution. The lowest CLA concentration was recorded in the case of infusions prepared by weighing 0.6 g of raw material (50 mL H_2_O) and infusing for 10 min. These values were almost half compared to the highest concentration (*p* < 0.001). Statistically significant differences were found between the median CLA content in infusions prepared by weighing different portions of the raw material and infusing for 20 min (*p* < 0.001). Sperman’s correlation showed a significant correlation between the method of preparing water infusions and the content of chlorogenic acid in them (R = −0.72; *p* < 0.001). It was also noticed that there were statistically significant differences between the average CLA concentrations in infusions prepared from raw materials from different producers. The CLA content was approximately 8% higher for producer 3 compared to producer 1 (*p* = 0.013). Taking into account the manufacturer and the CLA content in the infusions, a significant correlation was noted (R = 0.31; *p* = 0.003). In the studies, the results were also expressed in terms of dry product. It was found that the average median CLA content for all products was 26.36 mg in 100 g of product.

Analyzing the method of preparing water infusions, the content of chlorogenic acid in water infusions decreased in the following order: 1.0 g of raw material with 50 mL of water, brewed for 10 min (extraction method A) > 1.0 g of raw material with 50 mL of water, brewed for 20 min (extraction method B) > 0.6 g of raw material with 50 mL of water, brewed for 20 min (extraction method D) > 0.6 g of raw material with 50 mL of water, brewed for 10 min (extraction method C).

The research also examined the total phenol content (TPC) in water infusions prepared from motherwort herb and compared it with the CLA content. Table 4 presents the results obtained for the total content of phenols in water infusions prepared from motherwort herb. The research shows that the TPC in all analyzed infusions was 13.15 mg/100 mL, and the range was 7.56–27.08 mg/100 mL. Calculated per glass, this value is 32.88 mg, and range was 18.91–67.69 mg/250 mL. Sperman’s correlation showed a significant correlation between the method of preparing water infusions and the content of phenolic compounds in them (R = −0.35; *p* = 0.001). As part of the conducted research, it was found that the highest concentration of TPC in the analyzed infusions was obtained in the case of extracts prepared by weighing 1 g of raw material (50 mL of water) and infusing for 20 min. Statistically significant differences were also found between the TPC content in water infusions prepared by weighing raw materials from producers 1 and 3 (*p* < 0.001) and producers 1 and 2 (*p* = 0.049). Spearman’s correlation showed a significant correlation between the producer and the content of phenols in the infusion (R = −0.54; *p* < 0.001). Calculated on dry weight, the median TPC in all analyzed motherwort products was 0.66 g/100 g.

Analyzing the method of preparing water infusions, the TPC in water infusions decreased in the following order: 1.0 g of raw material with 50 mL of water, brewed for 10 min (extraction method A) > 1.0 g of raw material with 50 mL of water, brewed for 20 min (extraction method B) > 0.6 g of raw material with 50 mL of water, brewed for 20 min (extraction method D) > 0.6 g of raw material with 50 mL of water, brewed for 10 min (extraction method C).

The study also compared the content of CLA versus TPC. It was found that for all analyzed infusions, chlorogenic acid constitutes approximately 4% of all polyphenolic compounds. Taking into account various methods of preparing infusions, the percentage range was 3.10–3.75%.

Infusions prepared by weighing 0.6 g of the raw material showed higher radical scavenging activity. Table 5 presents the antioxidant activity of water infusions prepared from motherwort herb. The mean median in the DPPH test expressed as Trolox equivalent was 33.65 mg/L TE, while the range was 33.27–34.31 mg/L TE. Interestingly, higher activity was obtained for infusions prepared by weighing 0.6 g of the raw material and infusing it for 20 min than in the case of an infusion prepared by weighing 1.0 g of raw material and brewing for 10 min (*p* = 0.058). It was also noticed that there were statistically significant differences between the percentage of inhibition DPPH (I%) in infusions prepared from raw materials from different producers. The I% was approximately 8% higher for producer 2 compared to producer 1 (*p* < 0.001) and 3 (*p* < 0.001). Also, Spearman’s correlation showed a strong and significant correlation between the manufacturer and the I% (R = −0.73; *p* < 0.001).

In the DPPH test, the antioxidant properties of water infusions prepared from motherwort herb decrease in the following order: 0.6 g of raw material + 50 mL H_2_O and 10 min of brewing > 0.6 g of raw material + 50 mL H_2_O and 20 min of brewing > 1.0 g of raw material +50 mL H_2_O and 10 min of brewing > 1.0 g of raw material +50 mL H_2_O and 20 min of brewing.

## 4. Discussion

Over the last 20 years, the interest in the consumption of polyphenolic compounds and their biological properties has made it necessary to estimate their consumption in nutritional and pharmaceutical sciences. Estimating phenol intake is not an easy task, mainly due to the biodiversity of plant material and the lack of a standardized analytical method methods enabling the analysis of all classes or compounds of polyphenols.

It was already determined in the 1980s that 40% of all flavonoids come from teas, non-alcoholic beverages, coffee, cocoa, wine and beer. Fruits, berries and fruit juices provide about 30% of these products [13]. The Phenol-Explorer Database provides the possibility of daily intake of compound phenolic substances, as well as individual phenolic fractions. Thanks to such tools, the phenol intake for Finnish adults was estimated to be approximately 863 mg/day and for French adults to be approximately 1193 mg/day [13]. In Poland, the estimated consumption of these compounds is approximately 989.3 mg [14]. However, it should be noted that the actual consumption of phenols may be even higher. Over the last 10 years, the databases have been constantly updated as knowledge progresses [15,16]. Therefore, this research focused on estimating the content of chlorogenic acid in water infusions prepared from motherwort herb. So far, there have been no works describing this issue.

Due to various possible methods of preparing motherwort herb [2,9,17], the study assessed the content of CLA in water infusions of motherwort herb. It was found that the median CLA content, regardless of the method of preparing the infusion, was 527.26 μg/100 mL (1.32 mg/250 mL). Koshovyi et al. [2] estimated that the CLA content in 50% motherwort tinctures is 76.87 ± 0.57 mg/100 g. In our own research, regardless of the method of preparing the infusions, the amount of CLA in 100 g of dry product was 26.36 mg, about 60% less than Koshovyi et al. In the case of preparing an infusion by weighing 1.0 g of raw material and infusing it for 10 min (50 mL), the CLA content was obtained at the level of 32.11 mg in 100 g of the product, which is close to half of the values obtained by Koshovyi et al. It is estimated that ethanol extracts of *Leonurus cardiaca* are a richer source compared to aqueous ones. This is also confirmed by the research of Angeloni et al. [18]. Crude ethanol extracts prepared from aerial parts of *Leonurus cardiaca* picked in Poland contained 2.194 and 2.779 mg/g. While the purified ethanol extracts obtained using adsorption resins contained 4.012 and 6.367 mg/g of CLA [18]. Similar results were obtained by Bernatoniene et al. [17], which indicate that the CLA concentration in the 70% ethanol extract *Leonurus cardiaca* extract was 37.5 ± 4.0 µg/mL [17]. In our own research, the results were approximately 84% lower than in the research of Bernatoniene et al. Research by Zhogov et al. [9] shows that 50% ethanol extract of *Leonurus quinquelobatus* contains CLA in the range of 0.34–3.20 mg/g [9]. In our own research, the CLA content in water infusions prepared from the dried herb of the motherwort *Leonurus cardiaca* ranges from 0.10–0.45 mg/g. Similar results were obtained in the study by Safra et al. [19] using capillary isotachophoresis to analyze the CLA content in *Melissa herba*. It was found that the CLA content in *Melissa herba* methanol extract is approximately 0.30 mg/g [19]. Zhou et al. showed that the average CLA content in methanol extracts of *Acanthopanax senticosus* leaves was 1.7 mg/g [20]. It should also be noted that infusions prepared from 2.0 g of fresh *Crataegus pinnatifida* fruit called ‘Shanzha’ and infused for 5, 10 and 15 min in 100 mL of boiling water contain 0.919, 0.968 and 0.998 mg/g of methanol extract [21]. In our own research, preparing an infusion by weighing 1.0 g of motherwort herb and roasting it for 10 min at 100 °C led to results that were three times lower. For comparison, in green Arabica and Robusta beans, the CLA content can range from 0.98 (vary dark) to 45.34 (green) mg/g [7].

Consuming one glass (250 mL) daily contributes to a CLA intake of 1.32 mg/day. According to manufacturers’ recommendations, drinking two or three glasses a day increases CLA intake to 2.64 or 3.96 mg/day. The daily intake of phenols with one glass of infusion is 32.88 mg, with two glasses, 65.76 mg, and with three glasses, less than 100 mg. Our own research shows that 1 g of *Leonurus cardiaca* contains phenols in the range of 5.63–9.42 mg, depending on the method of preparing the infusion. However, it should be noted that extracts prepared using organic solvents are a richer source of phenols [22]. The use of hexane, diethyl ether, ethyl acetate and isopropyl alcohol as extractants resulted in a phenol content of 30.3, 48.2, 55.6 and 68.8 mg/g DW. In turn, the methanol–water extract (80:20) prepared from aerial parts of *Leonurus cardiaca* contains 42.95 mg GAL/g [23]. Similar results were obtained by Sadowska et al., which indicate that the acetone–water (70:30) extract prepared from motherwort herb has 137.7 mg GAL/g [24], with results about 13 times higher than for water extracts (Table 5). Motherwort herb, which was obtained from an orchard farm in Ozierany Małe in Podlasie, was tested for the antioxidant properties of water extracts prepared by weighing 50 g of the herb and pouring 1000 mL of water at 90 degrees (infusion time: 15 min) [25]. The results indicate that these infusions are a seven-fold richer source of phenols than in our own research. This is due to the difference in the preparation of the infusions. In the research of Telichowska et al. [25], the ratio of the weighed raw material to the used water was 5:100, and in our own research it was 2:100. However, it should be noted that in the same study, the phenol content in *Achillea millefolium* extracts was 26.65 mg GAL/g, which is approximately three times higher than in *Leonurus cardiaca* extracts prepared by weighing 1.0 g of motherwort herb and infusing it for 10 min in 50 mL of water (100 °C).

Research has shown that the analyzed aqueous infusions prepared from products containing dried motherwort herb are a source of compounds with anti-radical and antioxidant properties. Regardless of the method of preparing the infusions, in our own research, the antiradical effects using the DPPH test were comparable. The average activity for aqueous infusions was found to be 33.65 mg/L TE. Preparation of water infusions of motherwort herb in a ratio of 5:100, using the method of Telichowska et al. [25], resulted in the activity of water extracts at the level of 3.32 mg/L TE, which is almost 10 times higher than in our own research. The results obtained in our own research are comparable to those of other authors. According to the literature data, the use of organic solvents is not effective for the extraction of phenols [23]. The % DPPH inhibition of methanol–water extracts is about 85.84%. In turn, in our own research, water extracts of motherwort herb showed comparably strong antioxidant properties, as the %I range for four different infusions was 81.05–83.41%.

## 5. Conclusions

The method of preparing the infusions influenced the content of chlorogenic acid and phenols. However, it did not significantly affect the antioxidant properties of the prepared extracts. Preparing an infusion by weighing 1.0 g of motherwort herb and brewing it for 20 min in 50 mL of water results in an almost twice as high increase in CLA compared to an infusion prepared by weighing 0.6 g of the raw material without changing the brewing method. Moreover, extending the infusion time by 100% in the case of infusions prepared from weighing 1.0 g of raw material did not result in an increase in the amount of CLA in the water infusion.

Research indicates the need for systematic determination and monitoring of CLA content in water infusions from plant raw materials to increase the possibility of estimating the daily intake of chlorogenic acid with a varied diet. First of all, it should be emphasized that the presented results can be used to expand databases used by dietitians, doctors and pharmacists. Moreover, these studies provide information on the consumption of CLA with motherwort infusions, which may help in planning clinical trials on the effects of aqueous extracts of motherwort in in vitro studies, e.g., the effect of CLA on gastrointestinal transport and the pharmacological effects of different doses of chlorogenic acid.

## Figures and Tables

**Table 1:** Manufacturers’ recommendations regarding the method of preparation and frequency of consumption of infusions prepared from dried motherwort herb.

M	Method of Preparing the Infusion According to the Manufacturer’s Recommendations	Daily Consumption
1	Pour boiling water over it and set aside for 10 min	Nd
2	Pour 1 teaspoon of the herb into a glass (250 mL) of boiling water and set aside for 15–20 min 250 mL	3–4 x/day ¼ glass
3	Pour 1/2 cup (100 mL) of boiling water over 1 teaspoon of the herb and set aside for 20 min	2–3 x/day 1 glass

Nd—no data; M—manufacturer.

**Table 2:** Images of (aerial parts or some other parts) of *L. cardiaca* from different manufacturers (Manufacture 1—Flos, Mokrsko, Poland; Manufacture 2—Natura Vita, Pińczów, Poland; Manufactrue 3—Herbapol, Kraków, Poland used in this research.

Manufacture 1	Manufacture 2	Manufacture 3
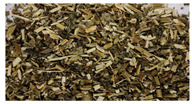	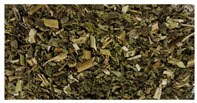	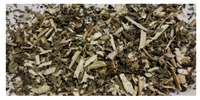

**Table 3:** Content of chlorogenic acid in different water infusions prepared from dried motherwort herb.

	M	Extraction Method (Me; Q_25_–Q_75_)				
		A	B	C	D	Total
CLA [µg/100 mL]	Total	642.12 ^ab^ (611.00–841.00)	624.01 ^cd^ (582.60–709.53)	349.15 ^ac^ (255.59–452.46)	405.13 ^bd^ (334.07–445.70)	527.26 (403.19–643.79)
	1	631.55 (593.91–636.48)	561.21 (526.51–615.75)	272.99 (235.41–339.83)	368.60 (262.79–405.13)	527.26 (403.19–643.79)
	2	752.21 (679.71–859.85)	720.73 (716.40–803.42)	319.21 (317.44–349.15)	404.79 (377.75–414.44)	564.25 (363.45–736.47)
	3	664.57 (584.50–841.99)	625.86 (619.50–709.27)	460.83 (420.50–643.01)	440.35 (422.50–518.01)	582.73 ^A^ (455.01–647.50)
CLA [mg/250 mL]	Total	1.60 ^ab^ (1.53–2.10)	1.56 ^cd^ (1.46–1.78)	0.87 ^ac^ (0.64–1.13)	1.01 ^bd^ (0.84–1.11)	1.32 (1.01–1.61)
	1	1.58 (1.48–1.59)	1.40 (1.32–1.54)	0.68 (0.58–0.85)	0.92 (0.657–1.013)	1.20 ^B^ (0.78–1.52)
	2	1.88 (1.69–2.15)	1.80 (1.79–2.01)	0.79 (0.79–0.87)	1.01 (0.94–1.04)	1.39 (0.91–1.84)
	3	1.66 (1.46–2.10)	1.54 (1.54–1.77)	1.15 (1.05–1.61)	1.10 (1.06–1.29)	1.53 ^B^ (1.14–1.62)
CLA [mg/100 g]	Total	32.11 ^ab^ (30.55–42.05)	31.20 ^cd^ (29.13–35.48)	17.46 ^ac^ (12.78–22.62)	20.56 ^bd^ (16.70–22.29)	26.36 (20.16–32.19)
	1	31.58 (29.70–31.82)	28.06 (26.33–30.79)	13.65 (11.77–16.99)	18.43 (13.14–20.26)	23.95 ^C^ (15.67–30.36)
	2	37.61 (33.99–42.99)	36.04 (35.82–40.17)	15.96 (15.87–17.46)	20.24 (18.89–20.72)	27.94 (18.17–36.82)
	3	33.23 (29.23–42.09)	31.29 (30.98–35.46)	23.04 (21.03–32.15)	22.02 (21.13–25.90)	30.62 ^C^ (22.75–32.38)

A—1.0 g of sample with 50 mL H_2_O (100 °C), 10 min; B—1.0 g of sample with 50 mL H_2_O (100 °C), 20 min; C—0.6 g of sample with 50 mL H_2_O (100 °C), 10 min; D—0.6 g of sample with 50 mL H_2_O (100 °C), 20 min. CLA—chlorogenic acid; M—manufacturer; Me—median; Q_25_—first quartile; Q_75_—third quartile; letters (a–d for values in rows; A–C for values in column) indicate statistically significant differences (*p* < 0.05).

**Table 4:** Content of total phenols content in different water infusions prepared from dried motherwort herb.

	M	Extraction Method (Me; Q25–Q75)				
		A	B	C	D	Total
TPC [mg/100 mL]	Total	17.14 ^a^ (11.85–20.30)	18.71 ^b^ (11.46–20.09)	11.23 ^ab^ (8.89–13.69)	11.94 (9.69–14.05)	13.15 (10.73–19.03)
	1	18.78 (17.14–20.09)	19.23 (18.71–19.91)	12.57 (10.59–13.64)	12.96 (11.82–13.27)	16.91 ^A^ (12.96–18.08)
	2	25.49 (25.18–25.52)	25.12 (24.98–25.18)	17.68 (16.17–18.27)	20.20 (18.07–20.66)	20.69^A^ (16.92–25.16)
	3	11.38 (10.67–11.87)	11.67 (10.77–11.45)	8.41 (7.79–9.51)	9.18 (8.55–9.71)	10.20 ^A^ (8.73–11.37)
TPC [mg/250 mL]	Total	42.85 ^a^ (29.63–50.75)	46.77 ^b^ (28.63–50.24)	28.08 ^ab^ (22.23–34.22)	29.84 (24.23–35.13)	32.88 (26.83–47.57)
	1	46.94 (42.85–50.24)	48.08 (46.77–49.78)	31.43 (26.49–34.10)	32.40 (29.56–33.19)	42.28 ^A^ (32.40–47.71)
	2	63.72 (62.94–63.79)	62.79 (62.44–62.87)	44.19 (40.43–45.68)	50.51 (45.18–51.64)	51.72 ^A^ (42.31–62.90)
	3	28.46 (26.68–29.67)	28.42 (26.93–28.64)	21.04 (19.47–23.77)	22.95 (21.39–24.27)	25.51 ^A^ (21.82–28.42)
TPC [g/100 g]	Total	0.86 ^a^ (0.592–1.015)	0.94 ^b^ (0.57–1.01)	0.56 ^ab^ (0.44–0.68)	0.58 (0.48–0.70)	0.66 (0.54–0.95)
	1	0.94 (0.85–1.01)	0.96 (0.95–0.99)	0.62 (0.53–0.68)	0.65 (0.59–0.66)	0.85 ^A^ (0.65–0.95)
	2	1.27 (1.26–1.28)	1.26 (1.25–1.26)	0.88 (0.81–0.91)	1.01 (0.90–1.03)	1.03 ^A^ (0.85–1.25)
	3	0.57 (0.53–0.59)	0.57 (0.53–0.57)	0.42 (0.39–0.48)	0.46 (0.42–0.49)	0.51 ^A^ (0.43–0.57)

A—1.0 g of sample with 50 mL H_2_O (100 °C), 10 min. (extraction method A); B—1.0 g of sample with 50 mL H_2_O (100 °C), 20 min (extraction method B); C—0.6 g of sample with 50 mL H_2_O (100 °C), 10 min (extraction method C); D—0.6 g of sample with 50 mL H_2_O (100 °C), 20 min (extraction method D). M—manufacturer; Me—median; TPC—total phenols content; Q_25_ —first quartile; Q_75_—third quartile; letters (a,b for values in rows; A for values in column) indicate statistically significant differences (*p* < 0.05).

**Table 5:** Antioxidant properties of different water infusions prepared from dried motherwort herb.

	M	Extraction Method (Me; Q25—Q75)				
		A	B	C	D	Total
DPPH [mg/L TE]	Total	33.50 (32.45–36.01)	33.27 (32.58–33.83)	34.31 (32.70–36.05)	34.07 (32.25–34.62)	33.65 (32.68–34.94)
	1	33.27 (33.10–33.62)	33.34 (32.99–33.52)	34.19 (33.65–34.35)	34.10 (33.97–34.21)	33.65 (32.68–34.94)
	2	36.04 (36.01–36.09)	32.25 (31.96–34.01)	36.21 (36.04–36.37)	32.98 (32.25–33.23)	33.64 (33.19–34.14)
	3	32.92 (32.15–35.59)	33.21 (32.62–35.11)	33.76 (31.21–35.78)	35.49 (31.72–36.56)	33.33 (32.05–35.64)
DPPH [mM TE]	Total	134.05 (129.66–143.85)	132.92 (130.15–135.15)	137.09 (130.64–144.01)	136.12 (128.85–138.33)	134.46 (130.56–139.61)
	1	132.93 (132.25–134.32)	133.22 (131.83–133.91)	136.60 (134.46–137.22)	136.26 (135.70–136.67)	134.46 (130.56–139.61)
	2	144.01 (143.85–144.18)	128.85 (127.71–135.86)	144.66 (144.01–145.32)	131.78 (128.85–132.76)	134.39 (132.59–136.39)
	3	131.53 (128.44–142.22)	132.68 (130.31–140.26)	134.88 (124.69–142.95)	141.81 (126.73–146.05)	133.17 (128.03–142.38)
DPPH [I%]	Total	82.89 81.19–86.22)	81.05 (76.55–86.30)	83.41 (76.77–88.27)	83.04 (77.29–88.11)	83.03 (77.43–86.75)
	1	86.22 (85.81–87.04)	86.38 (85.56–86.79)	88.39 (87.12–88.76)	88.19 (87.86–88.43)	83.03 ^A^ (77.43–86.75)
	2	82.81 (82.74–82.89)	75.96 (75.44–79.13)	83.11 (82.82–83.41)	77.29 (75.96–77.73)	87.08 ^A^ (86.01–88.27)
	3	77.18 (75.77–82.01)	77.69 (76.62–81.12)	78.69 (74.08–82.34)	81.82 (75.01–83.74)	77.91 ^A^ (75.59–82.08)

A—1.0 g of sample with 50 mL H_2_O (100 °C), 10 min; B—1.0 g of sample with 50 mL H_2_O (100 °C), 20 min; C—0.6 g of sample with 50 mL H_2_O (100 °C), 10 min; D—0.6 g of sample with 50 mL H_2_O (100 °C), 20 min DPPH—2,2-difenylo-1-pikrylohydrazyl. M—manufacturer; Me—median; TE—Trolox, (+-)-hydroxy-2,5,7,8-tetramethyl-chroman-2-carboxylic acid; Q_25_—first quartile; Q_75_—third quartile; letter A indicate statistically significant differences between values in column (*p* < 0.05).

## Data Availability

The original contributions presented in this study are included in the article. Further inquiries can be directed to the corresponding author.

## Abbreviations

BGE: background electrolyte (20 mM Tetraborate buffer pH = 9.4); CLA: chlorogenic acid (3-O-Caffeoylquinic acid); CV Precision: coefficient of variation precision; DPPH: 2,2-diphenyl-1-picrylhydrazyl; ETOH: ethyl alcohol; FC: Folin–Ciocâlteu phenol reagent; GAL: gallic acid (3,4,5-Trihydroxybenzoic acid); LOD: limit of detection; LOQ: limit of quantification; RSD: relative standard deviation; STD: standard deviation; TE: Trolox equivalent ((+-)-hydroxy-2,5,7,8-tetramethyl-chroman-2-carboxylic acid); TPC: total phenol content.
